# “*Who has to do it at the end of the day? Programme officials or hospital authorities?*” Airborne infection control at drug resistant tuberculosis (DR-TB) centres of Karnataka, India: a mixed-methods study

**DOI:** 10.1186/s13756-017-0270-4

**Published:** 2017-11-06

**Authors:** Kibballi Madhukeshwar Akshaya, Hemant Deepak Shewade, Ottapura Prabhakaran Aslesh, Sharath Burugina Nagaraja, Abhay Subashrao Nirgude, Anil Singarajipura, Anil G. Jacob

**Affiliations:** 1 Department of Community Medicine, Yenepoya Medical College, Yenepoya University, Mangaluru, 575018 India; 2 International Union against Tuberculosis and Lung Diseases, South East Asia Office, New Delhi, India; 3 Department of Community Medicine, Government Medical College, Thrissur, India; 4 Department of Community Medicine, ESIC Medical College and PGIMSR, Bengaluru, India; 5 Department of Health and Family Welfare, Government of Karnataka, Bengaluru, India

**Keywords:** Infection control, Drug-resistant tuberculosis, MDR tuberculosis, Hospital infections, SORT IT

## Abstract

**Background:**

Drug resistant tuberculosis (DR-TB) centers admit patients with DR-TB for initiation of treatment and thereby concentrate the patients under one setting. It becomes imperative to assess the compliance of DR-TB centres to national airborne infection control (AIC) guidelines and explore the provider perspectives into reasons for unsatisfactory compliance.

**Methods:**

This mixed methods study (triangulation design) was carried out across all the six DR-TB centers of Karnataka state, India, between November 2016 and April 2017. Non-participant observation using a structured format was carried out at the DR-TB wards (*n* = 6), outpatient departments (*n* = 6), patient waiting areas outside outpatient departments (*n* = 6) and culture and drug susceptibility testing laboratories (*n* = 3). Structured interviews of admitted patients (*n* = 30) were done to assess the knowledge on cough hygiene and sputum disposal. Key informant interviews (KIIs) of health care providers (*n* = 20) were done. Manual descriptive content analysis was done to analyse the transcripts of KIIs.

**Results:**

The findings related to compliance in non-participant observation were corroborated by KIIs. All the laboratories were consistently implementing the AIC guidelines. Compliance to hand hygiene, wet mopping and ventilation measures were satisfactory in four or more DR-TB wards. The non-availability of N95 masks in wards as well as outpatient departments was staggering. Sputum disposal without prior disinfection and the lack of display materials on cough hygiene and patient education was common. Patient fast tracking in outpatient department waiting areas and visitor restrictions in wards were lacking. Trainings on AIC measures were uncommon. About half and one-third of patients admitted had satisfactory knowledge regarding sputum disposal and situations demanding mask respectively. The reasons for unsatisfactory compliance to AIC guidelines were poor coordination between programme and hospital authorities leading to lack of ownership; ineffective or non-existent infection control committees; vacant posts of medical officers; and attitudes of health care delivery staff.

**Conclusion:**

Compliance with AIC guidelines in DR-TB centers of Karnataka was sub-optimal. The reasons identified require urgent attention of the programme managers and hospital authorities.

**Electronic supplementary material:**

The online version of this article (10.1186/s13756-017-0270-4) contains supplementary material, which is available to authorized users.

## Background

Despite Tuberculosis (TB) being a curable disease through the use of standardized drugs, the increasing resistance to these drugs in the form of multi-drug resistant tuberculosis (MDR-TB), defined as resistance to isoniazid and rifampicin or rifampicin only, is a growing public health problem across the globe. India has the highest burden of MDR-TB patients in the world. An estimated 130,000 MDR-TB patients occur annually in India which includes 79,000 estimated among notified pulmonary cases [1, 2]. India follows programmatic management of drug resistant TB (PMDT) guidelines within the revised national TB control programme (RNTCP) [3]. These services have expanded across all the states and union territories of India covering the whole population by 2013 [2].

Under PMDT, patients with MDR-TB are treated primarily on domiciliary basis after a brief period of in-patient care during treatment initiation in Drug Resistant TB (DR-TB) centers (one for every 10 million population). These are located within the premises of a medical college hospital or a tertiary public health facility (catering to patients other than MDR-TB as well), under the auspices of departments of pulmonary medicine or internal medicine (if the former does not exist). Each DR-TB centre has a separate ward for male and female patients, PMDT services [culture and drug susceptibility testing (CDST) and ancillary drugs for management of adverse drug reactions] and availability of relevant specialties or linkages for these services established [3]. Currently there is a network of 143 DR-TB centres across the country which are supported by 54 linked DR-TB centres (decentralized clinical unit under a DR-TB centre which provides treatment services but reporting lies with the parent DR-TB centre) [2].

In India, the treatment approach for patients with DR-TB concentrates the drug resistant patients under one roof during the initiation of treatment and also during the management of complications. Patients with MDR-TB in these settings pose a potential source of infection to the health care providers and other patients in these facilities who may be immune-compromised [4–7]. The transmission of the infection can be reduced by implementing effective airborne infection control measures in these settings [8, 9].

The national airborne infection control (AIC) guidelines were drafted in 2010 which proposed standards for AIC across various health facilities including high risk settings like MDR-TB wards [10]. A study assessing the implementation of these guidelines which was carried out across 35 healthcare facilities (these included four MDR-TB wards) in three states of India revealed that AIC practices were poorly implemented [11]. After rapid expansion of the PMDT services in the country there is scarce information on implementation of these guidelines at DR-TB centres and the barriers faced by the health care providers in complying with these guidelines.

Hence, a study was conducted to assess the compliance of DR-TB centres to National AIC guidelines (2010) and explore the provider perspectives related to the barriers encountered for implementation in the state of Karnataka, India (2016-17).

## Methods

### Study design

It was a cross-sectional study involving a mixed-methods approach (triangulation design) where compliance to the implementation of AIC guidelines were assessed through non-participant observation of DR-TB centers, structured interviews of admitted patients and key informant interviews (KIIs) of health care providers [12].

### Study setting

#### General setting

Karnataka is the eighth largest state of India in terms of both area and population and is situated on the western edge of the Deccan plateau. The state has an area of 191,791 sq. km. and a population of 61.1 million [13]. It is divided into 30 administrative districts.

#### DR-TB centres

**Fig. 1:**
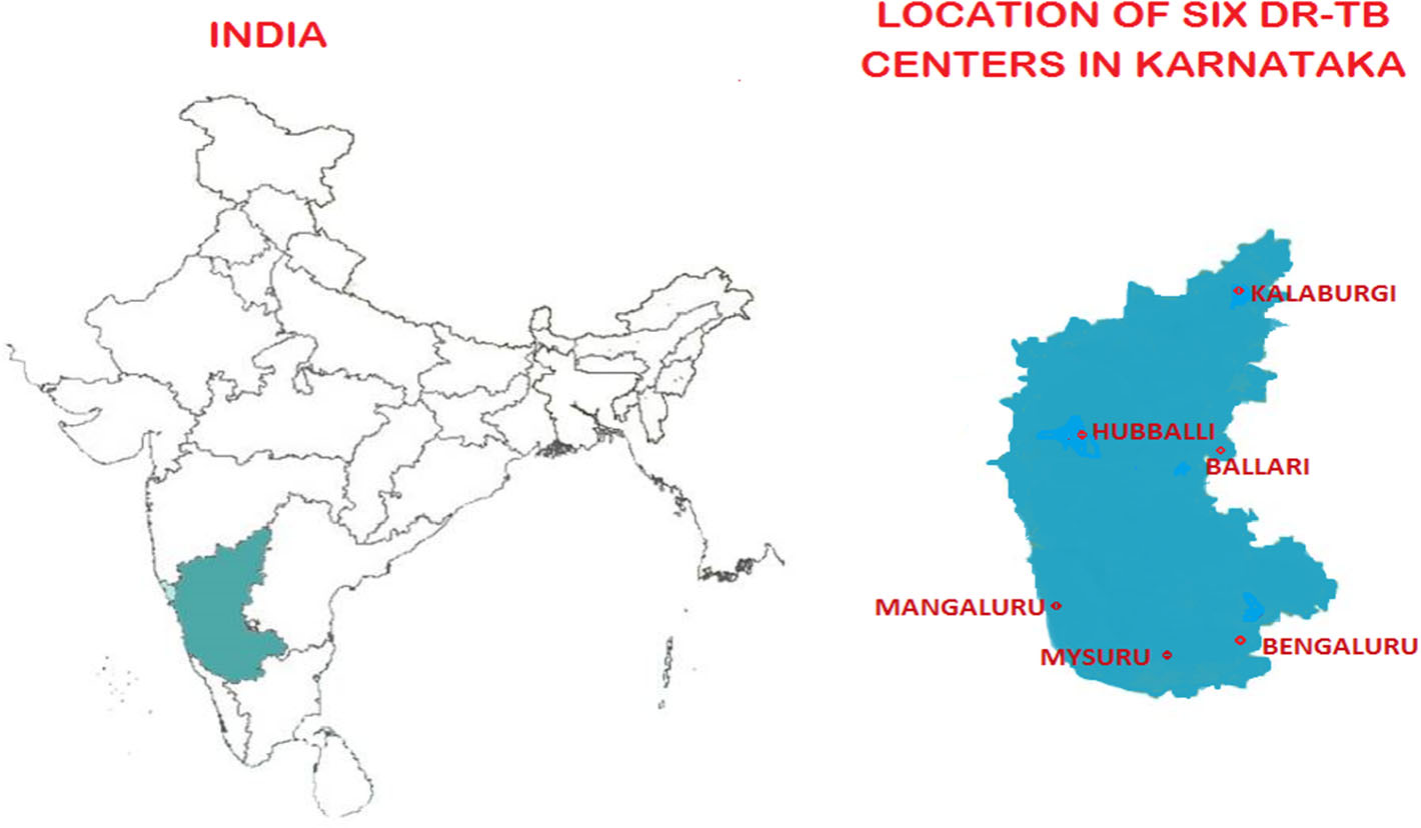
The six drug resistant TB (DR-TB) centres in the state of Karnataka, India (2016-17)

PMDT services in Karnataka are delivered through six functional DR-TB centres situated at Bengaluru, Mysuru, Mangaluru, Hubballi, Ballari and Kalaburgi (Fig. 1). Patients with TB detected with rifampicin resistance are registered for MDR-TB treatment. Patients undergo second-line DST of patients with MDR-TB fit the presumptive extensively drug resistant TB criteria. A total of 1338 patients were detected with MDR-TB and 1099 of them were registered and initiated on treatment in these centres in 2016 [2, 3].

DR-TB centers are located in tertiary public healthcare facilities known as TB and chest diseases hospitals (Bengaluru, Mysuru, Ballari) as well as government medical college/district hospitals (Hubballi, Kalaburgi, Mangaluru). These centers were set up with one-time provision of up to USD 15500 from RNTCP for up-gradation of the existing wards to incorporate airborne infection control measures. The DR-TB centers are administratively controlled by the health of the institution (director or medical superintendent - administrator of these tertiary public health facilities/ medical college/ district hospitals). The routine health care services are provided by a specialist working in pulmonary medicine or internal medicine department (nodal officer), nursing and housekeeping staff. The national TB control programme supports the DR-TB centers by providing drugs and additional human resources that include a medical officer, a counsellor and a statistical assistant. However, the routine clinical laboratory investigation facilities for pre treatment evaluation and monitoring, ancillary drugs as per DR-TB centre committee’s advice, drugs required for management of adverse drug reactions should be made available from the institute as part of their commitment for which no reimbursement will be available from the programme [3].

### Study population

All the six DR-TB centers in Karnataka, India, were included in the study. These contained 21 units for AIC assessment: chest medicine/ internal medicine out-patient department (OPD) (*n* = 6), waiting areas outside the OPDs (*n* = 6), DR-TB wards (*n* = 6) and CDST laboratories (*n* = 3). All the in-patients admitted in the DR-TB wards at the time of visit by principal investigator (PI) drawn from the line list prepared by the staff nurse on duty were also interviewed.

Health care providers across four different cadres involved in providing DR-TB care at these facilities were interviewed (*n* = 20). We intended to interview all the administrators and medical officers of the DR-TB center. In the absence of the medical officer, the nodal officer was interviewed subject to availability. The nursing and the housekeeping staff who were on duty at the time of visit of the PI were interviewed based on convenience sampling.

### Data collection, variables and sources of data

Data collection was done between November 2016 and April 2017. A checklist (dichotomous variables) drawn from the national AIC guidelines [10] was administered through non-participant observation (Additional file 1: Annex S1) and in-patients with MDR-TB were interviewed about cough hygiene and sputum disposal using a structured closed ended interviewer administered questionnaire [translated into the local language (Kannada), back translated for content validation and pretested at Mangaluru]. (Additional file 1: Annex S2) Systematic qualitative enquiry was done through key informant interviews of health care providers which included administrators (*n* = 4), medical officers or nodal officers (*n* = 4), staff nurses (*n* = 6) and housekeeping employees (*n* = 6). Separate interview guides were prepared for these different cadres of health care staff and further modified based on the results of our non-participant observations and pre-testing at Mangaluru. (Additional file 1: Annex S3).

The interviewer, a 36 years old male (KMA), a faculty member (M.B.B.S; M.D.; D.N.B.) in a medical college in the region was trained in the use of qualitative research methods. The interviews were conducted in English or local language (Kannada) at their workplace after obtaining their written permission and consent to participate in the study. Administrators and medical officers or nodal officers were a priori informed over phone about the purpose of visit and their expected role in the interview. Only the participant and the researcher were present during the interview. Depending on participant comfort, consent was sought for audio recording; verbatim written notes were taken during the interview. The duration of the interviews varied between 5 and 20 min.

### Data entry and analysis

Quantitative data collected were double-entered, validated and analysed using EpiData (version 3.1 for entry and version 2.2.2.183 for analysis, EpiData Association, Odense, Denmark). Frequency and proportions were used to summarise the key analytic outputs.

The interviews were transcribed and translated (Kannada to English), if required, within 48 h based on the notes and audio records of the interviews (KMA). These transcripts were compiled and read to become familiar with the data. Manual descriptive content analysis was done to analyse the transcripts. Categories were decided a priori and codes were generated inductively [12, 14]. These were reviewed by the second investigator (HDS) to reduce bias, increase inter-coder reliability and enhance interpretative credibility. The decision on coding rules and theme generation was done by using standard procedures and based on consensus [15]. Differences if any were resolved by discussion. Similar codes were combined into themes [12]. To ensure that the results are a reflection of the data, the codes/themes were related back to the original data [16]. While representing the quotes, square brackets within quotes were used to mention authors’ comments and round brackets were used to mention the sex and occupation of the participant. The findings were reported by using ‘Consolidated Criteria for Reporting Qualitative Research [17].

## Results

### Compliance to implementation of AIC guidelines

The findings of non-participant observation of the six DR-TB centers are summarised in Table 1. In DR-TB wards (*n* = 6), compliance to hand hygiene, wet mopping and ventilation measures were satisfactory in four or more wards. All other components were found unsatisfactory in five or more wards. Visitor restriction and adherence to the use of personal protective equipment were not implemented in any of the wards.

**Table 1:** Compliance to Airborne Infection Control guidelines among the DR-TB Centers in Karnataka, India, 2016-17 (*n* = 6)

Sl. No	Facility assessed	AIC guidelines	Components of assessment	Number of centers with satisfactory compliance to AIC guidelines (*n* = 6)
1	DR-TB Ward	Administrative	Location and design	1
			Visitor restriction	0
			Hand hygiene	4
			Cough hygiene	1
			Wet mopping	4
			Human resources training	1
		Environmental	Ventilation	5
		Personal Protective	Personal Protective Equipment	0
2	Patient waiting area	Administrative	Cough hygiene	0
			Patient fastracking	1
			Wet mopping	4
		Environmental	Ventilation	6
3	Chest medicine/ internal medicine Out Patient Department	Administrative	Hand hygiene	6
			Wet mopping	4
			Human resources training	3
		Environmental	Ventilation	5
		Personal Protective	Personal Protective Equipment	0
4	CDST Laboratory (n = 3)	Administrative	Standard Operating Procedures	2
			Human resources training	3
			Signage	2
			Lab reports	2
			Bio-safety checklist	2
			Hand hygiene	3
			Location and design	3
			Restricted entry	3
			Sterilization	3
		Personal Protective	Personal Protective Equipment	3

*AIC* airborne infection control, *DR-TB* drug resistant tuberculosis, *CDST* culture and Drug Sensitivity testing

All the patient waiting areas were adequately ventilated; however, patient fast tracking and cough hygiene components were unsatisfactory across the DR-TB centers. In the out-patient departments across the DR-TB centers, hand hygiene and ventilation components were satisfactory; however, the use of personal protective equipment for the health care providers was not implemented.

All the three CDST laboratories were consistently implementing all the AIC guidelines. Some exceptions were seen in the implementation of standard operating procedures, timely availability of lab reports and bio-safety checklist in one laboratory.

About three fourths of the patients admitted in the DR-TB wards had a satisfactory knowledge of cough hygiene. Half and one third of the patients knew about the appropriate method for disposal of sputum and situations demanding mask usage respectively (Table 2).

**Table 2:** Knowledge about Cough Hygiene and Sputum Disposal of the patients admitted in DR-TB wards of DR-TB centers, Karnataka, India, 2016-17 (*n* = 30)

Sl. No	Knowledge about the variables	Satisfactory knowledge n (%)
1	Cough hygiene	23 (76.7)
2	Purpose of use of surgical masks	21 (70.0)
3	Situations that demand mask usage	09 (30.0)
4	Disposal of sputum	16 (53.3)

KIIs confirmed the satisfactory implementation of wet mopping and adherence to hand hygiene. Two DR-TB wards had AIC compliant buildings and in the remaining four wards, existing hospital wards were structurally modified for in-patient treatment of DR-TB patients. We found that every patient was provided with a surgical mask on admission and educated about the seriousness of the illness, its transmission risk to others and cough hygiene.

The gaps in the effective implementation of AIC guidelines across administrative, environmental and personal protective categories have been summarised in Table 3. There were seven codes identified under these categories: lack of AIC compliant buildings, lack of visitor restriction, poor hand hygiene, unsafe sputum disposal, lack of training on AIC guidelines among staff, poor cross ventilation and lack of N95 mask.

**Table 3:** Gaps in the implementation of airborne infection control guidelines as perceived by the health care providers at DR-TB centers, Karnataka, India, 2016-17

Categories	Codes/ Themes	Verbatim quotes
Administrative AIC measures	Lack of AIC guidelines compliant buildings	“There are patients with other illnesses in the same building just separated by few meters and a wall” (male doctor) “The wards are located too closely with the wards housing patients from other departments” (male doctor)
	Lack of visitor restriction	“Usually there will be one attender or sometimes two staying with the patient. Even when we tell about the disease and how it spreads, they tell that it is difficult to stay away.” (female nurse) “As per our hospital policy, all the patients need to have one attender compulsorily with them at all the times.” (female nurse)
	Poor Hand Hygiene	“When it comes to washing, there are no soaps provided for hand washing. Sinks are put up but no water is available. Running water is required; however, it is not there.” (male nurse)
	Unsafe sputum disposal	“Actually, each patient has to spit the sputum in the sputum cup prefilled with some water. The contents are poured in to the toilet and flushed.” (female nurse) “There is presently no adequate supply of disinfectants; it may be phenol or even bleaching powder.” (female housekeeping staff) “The patients are told to spit the sputum in the washroom and flush it with adequate water.” (female housekeeping staff)
	Lack of training on AIC guidelines among staff	“I have not undergone any training. I have joined here just one year back.” (female housekeeping staff) “I have not received any sort of training before getting posted here. Nobody who is posted here receives any sort of training.” (female nurse) “There is attrition among the housekeeping staff and hence, a lot of new people are added to the pool regularly. Educating these people is also a challenge for us.” (male doctor) “Due to administrative policy of the hospital, they [nurses] are relocated to different wards once in 2 months.” (male doctor)
Environmental AIC measures	Poor cross ventilation	“Many a times at least during the nights, the windows are closed citing mosquito menace.” (male doctor)
Personal Protective AIC measures	Lack of N95 masks	“We are trying our best to provide the N95 masks. However, we don’t have supply at all times.” (male doctor) “I don’t feel very safe working in this ward and always have the fear of contracting the infection (because of lack N95 mask).” [female nurse] “I use mask when I am working in this ward and also in other departments of the hospital. This cloth mask!” (female nurse) “I am not using N95 mask as there is no supply. But what to do? They are costly (to be purchased).” (male doctor)

*DR-TB* drug resistant tuberculosis

### Reasons for unsatisfactory compliance

The reasons for unsatisfactory compliance to AIC guidelines along with relevant quotes across health system and individual levels are summarized in Table 4. We identified four codes: poor coordination between RNTCP and general health services leading to lack of ownership; ineffective or non-existent infection control committees; vacant posts; and attitudes of health care delivery staff.

**Table 4:** Provider perspectives into barriers for implementation of airborne infection control guidelines at DR-TB centers, Karnataka, India, 2016-17

Categories	Codes/ Themes	Verbatim quotes
Health system level	Poor coordination leading to lack of ownership	“The programme gives us a one-time grant to set up the ward and other required modifications; however there is no supply of consumables [ex. masks and disinfectants] by the programme at any point of time.” (male doctor) “We are not directly involved in the implementation of the TB prevention programme. We supplement it as a part of the teaching institution attached to that.” (male doctor) “N95 mask has to be supplied to all the medical and para medical staff by the hospital or program. There should be some display material provided by the program regarding cough hygiene and nutrition.” (male doctor) “We have placed an indent for these items required many a times but they are not supplied. We have even brought it to the notice of the district programme officials; however, it was told that it is the responsibility of the hospital to provide these.” (male doctor) “As per my rough estimate, we require about 300 odd N95 masks a month if it has to be used properly among all cadres of staff. Who has to supply this when each costs atleast a dollar per piece? There is always a tussle going on between the programme officials and the hospital authorities regarding the resources. The problem lies in “who has to do it at the end of the day?” (male doctor)
	Ineffective or non-existent Infection control (IC) committees	“We don’t have any regular meetings of the IC Committee as such.” (male doctor) “AIC of DR-TB wards are not addressed separately and no special measures are taken for AIC in these wards.” (female doctor) “They [housekeeping staff] really don’t know about personal protection. Most of them use after seeing us wearing the masks.” (female nurse) “There are also no set protocols and guidelines for the trainings.” (male doctor) “We don’t have a microbiologist required for the functioning of the IC committee” (male doctor)
	Vacant posts	“The medical officer post is vacant here. There is an acute shortage of staffs, only few nurses against the sanctioned number.” (male doctor) “Medical officers get trained and they leave after working here for some time and getting an experience.” (male doctor) “Medical officers are recruited on contractual basis and are poorly paid. There is no risk allowance for working in DR TB center.” (male doctor)
Individual level	Attitudes of health care delivery staff	“I don’t think exhaust fans are useful. The number of patients are very much less here, may be around 8-10 patients at the maximum. In that case for infection control, we don’t need these things.” (male doctor) “N95 masks are available only for the doctors; cloth masks are made available for the nurses and housekeeping staff.” (male doctor) “I don’t feel any risk to the nursing staff. None amongst us have suffered from any type of Tuberculosis.” (male nurse)

*DR-TB* drug resistant tuberculosis

## Discussion

This study is one of the few studies conducted in India to determine the implementation status of national AIC guidelines at DR-TB centres. The level of compliance with AIC guidelines among the DR-TB centers was sub-optimal when compared to the laid down guidelines (except for the CDST laboratories) at DR-TB wards, OPDs and patient waiting areas. As beneficiaries of the public health programme, there was scope for improving the existing patient knowledge with regard to cough hygiene. The findings related to compliance by non-participant observation were corroborated by KIIs. The recurring theme that emerged was a lack of investment and ownership as well as poor coordination between the national programme (RNTCP) and general health care system in the state as the main reason for unsatisfactory compliance.

### Strengths and limitations

Operational research is essential to develop evidence-based policies to improve TB IC [9]. This is the first comprehensive study on the state-wide assessment of the AIC measures in the DR-TB centers in India. This assessment is not routinely done within the programmatic context; therefore it can serve as a baseline study for future research. There was a convergent validity between the findings of the quantitative and qualitative components. This makes our findings robust.

There were some limitations of the study. First, we were not able to get an insight into the pragmatic solutions to address the reasons for unsatisfactory compliance. The staffs were not forthcoming with solutions. It could be because the nursing and housekeeping staffs were not informed a priori about the purpose of the visit. Second, an objective assessment of ventilation using appropriate tools for air flow and air velocity was not carried as we did not have the required scientific and allied resources.

### Interpretation of key findings

A key finding in this study is that discrepancies between guidelines, standards and programme implementation with respect to operating processes and procedures can be traced to ambiguities at higher levels owing to a short-term approach to institutional funding and design. These ambiguities percolate down through budgeting, hiring and contracting procedures. These asymmetries are palpable at multiple levels. At the scientific level, the variation of attitudes among doctors and nursing staff is one such asymmetry. At the level of human resource and staffing, short-term contracts compounded by low pay and lacking incentives to retain staff is another asymmetry. At the level of sensitizing staff, the lack of orientation sessions and on-going trainings is another. Finally, at the level of basic consumables, the lack of translating guidelines into budget-supported resources is yet another.

The N95 mask is one of the most important preventive measures for frontline health workers working in a high risk setting and is expected to reduce or even break the transmission cycle [10, 18]. Unfortunately, the non-availability of N95 masks in wards as well as OPDs was staggering. They were not available as a rule and if available, they were not provided to the nursing or housekeeping staff. The consistent non-availability of N95 masks was also pointed out in another study from South Africa [19].

On the side of patients, they were encouraged to dispose sputum either in the sink or the toilet without prior disinfection, posing a risk for transmission of MDR-TB, as observed in our study. At a pan-centre level, the lack of display materials on cough hygiene and patient education were not found in the wards and OPD waiting areas in most of the centers. These display boards act as reinforcement for health promoting behaviour and are a low-cost, high-impact way of educating multiple stakeholders [10].

Patient fast tracking in OPD waiting areas and visitor restrictions are other obvious measures which minimise the exposure of susceptible people to MDR-TB. These practices were not implemented in our setting. This is a major finding. It almost destroys the purpose of having a separate DR-TB ward. Finally, no policy or guideline can be successful without inviting the intelligent investments of key implementing stakeholders: the participants in our study reported that they have not undergone any training on AIC measures. Considering the frequent shifting of the nurses (which is a policy of the hospitals) and attrition among housekeeping staff; this is a vital point that needs to be addressed. Similar findings were reported in other studies from developing countries [11, 19, 20].

These gaps in AIC were contrary to one of the studies from South Africa where all the facilities assessed were found to have displayed signage to educate patients and health care workers on cough hygiene. N95 mask was uniformly available for use among the health care workers but they were not used. However, the ward visits by the visitors were a common practice similar to that in our study [21].

There were two main reasons for unsatisfactory compliance. First, there was poor coordination between RNTCP and general health care system (hospital authorities) leading to lack of ownership among the administrators, nodal officers and medical officers. After the one time grant by RNTCP to implement AIC guidelines in DR-TB centers, roles and responsibilities have been clearly demarcated across human resources. Though, it is clear from the national guidelines that the hospital authorities have to run the DR-TB center [3], there was lack of clarity regarding the onus of responsibility for financial resources, for acquiring the consumables such as N95 masks and phenol for sputum disinfection among others. The hospital authorities/ doctors did not consider it as their primary responsibility resulting in gaps in AIC. Vacant posts of medical officer (in four out of six DR-TB centers) could further contribute to this lack of ownership and also might be a deterrent for the on job training of the nursing and housekeeping staff in AIC measures. This is particularly important as the nodal officer is a specialist who also holds additional clinical and faculty positions and may not have the time for day to day on the job training and supervision of nursing and housekeeping staff.

Second, the IC committees were either non-existent or non-functional. Similar findings were observed in studies from India, South Africa and Pakistan [11, 19, 20]. However, another South African study reported that about 80% of the DR-TB centers assessed had IC committees [21]. Hospital IC committees are expected to perform risk assessment, prepare facility infection control plan incorporating the AIC component as well. IC committees need to have a realistic budget for carrying out and ensuring implementation of IC [10]. These activities including training of health staff were probably not being carried out resulting in gaps in AIC implementation. The use of simple videos in the local language can easily be rolled out to sensitize staff to how preventive measures are within reach. However, this is the responsibility of IC committees.

**Fig. 2:**
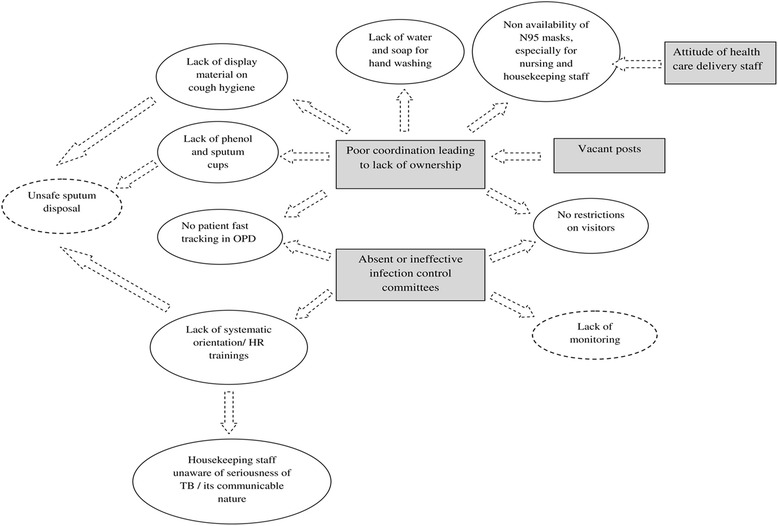
Visual framework of the barriers leading to gaps in implementation of AIC guidelines in DR-TB centers in Karnataka state, India (2016-17). DR-TB: Drug resistant tuberculosis; Square boxes indicate barriers, oval/circles indicate gaps, ovals with dotted line indicate author’s interpretation. Dotted line arrows are used to suggest possible causal linkages between themes

Based on the codes generated in our analysis (Tables 3 and 4) and the above interpretations, a visual framework of the reasons leading to gaps in implementation (unsatisfactory compliance) of AIC guidelines is depicted in Fig. 2.

### Recommendations for policy and practice

Based on our study findings, the following recommendations can be drawn. First, the administrators of the hospitals need to reinvigorate the existing infection control committees so that they perform their duties as per the suggested guidelines [10]. The programme needs to routinely incorporate the review of IC meetings held and AIC measures undertaken at DR-TB centers during the quarterly and national review meetings. Second, hospital authorities need to take the ownership of AIC in DR-TB centers and make budgetary allocations towards display of patient education on cough hygiene in prominent locations in the local languages and consumables like N95 masks, sputum cups and phenol for sputum disinfection. Third, standard operating procedures related to AIC for doctors, nurses as well as housekeeping staff should be displayed near their workstations in the local language. Fourth, vacant posts of Medical officers need to be filled up at the earliest by RNCTP and financial mechanisms need to be evolved to increase retention of the medical officers. Further research in deployment of newer technologies and equipments to measure and maintain airflow in the wards has to be prioritized.

## Conclusions

Compliance with AIC guidelines in the DR-TB centers of the state of Karnataka, India has a scope for improvement. The reasons for unsatisfactory compliance require urgent attention of the programme managers and hospital authorities, especially the latter, as the DR-TB centers are administratively under them. The gravity of MDR-TB (and the low cure rate of around 54% with the existing drug regimens) underlines the significance of taking long-sighted measures for a potentially dangerous disease that can be controlled with some investment of capital, administrative and scientific resources [22].

## Additional file

Additional file 1:
**Annex S1.** Operational definitions used for assessing compliance of DR-TB centers towards AIC measures in Karnataka, India, 2016-17. **Annex S2.** Operational definitions used to assess Knowledge about Cough Hygiene and Sputum Disposal of the patients admitted in DR-TB wards of DR-TB centers, Karnataka, India, 2016-17. **Annex S3.** Interview guides used for key informant interviews at Drug Resistant Tuberculosis (DR-TB) centres of Karnataka, India, 2016-17. (DOCX 25 kb)

## Abbreviations

AIC: Airborne infection control
CDST: Culture and Drug Sensitivity testing
DR-TB: Drug resistant tuberculosis
IC: infection control
KII: Key informant interview
MDR-TB: Multidrug resistant tuberculosis
OPD: outpatient department
PMDT: programmatic management of drug resistant TB
RNTCP: Revised national tuberculosis control programme
