# Supplementary material for: “Who has to do it at the end of the day? Programme officials or hospital authorities?” Airborne infection control at drug resistant tuberculosis (DR-TB) centres of Karnataka, India: a mixed-methods study
Source: Antimicrob Resist Infect Control. 2017 Nov 6;6:111. doi: 10.1186/s13756-017-0270-4 (PMC5674795; doi:10.1186/s13756-017-0270-4)
Supplement: Additional file 1: — Annex S1. Operational definitions used for assessing compliance of DR-TB centers towards AIC measures in Karnataka, India, 2016-17. Annex S2. Operational definitions used to assess Knowledge about Cough Hygiene and Sputum Disposal of the patients admitted in DR-TB wards of DR-TB centers, Karnataka, India, 2016-17. Annex S3. Interview guides used for key informant interviews at Drug Resistant Tuberculosis (DR-TB) centres of Karnataka, India, 2016-17. (DOCX 25 kb) [file 13756_2017_270_MOESM1_ESM.docx]

**Additional file 1**

**Annex S1:** Operational definitions used for assessing compliance of DR-TB centers towards AIC measures in Karnataka, India, 2016-17

| **Sl.No** | **AIC guidelines** | **Components of assessment** | **Operational definitions** |
| --- | --- | --- | --- |
| **1** | **DR-TB Ward** | | |
| 1.1 | Administrative AIC measures | Location and design | Considered satisfactory when MDR-TB ward is located away from other wards, there is a separate passage to toilets, the distance between two beds is at least 6 feet |
|  |  | Visitor restriction | Considered satisfactory when only one visitor is allowed to meet the patient during the stipulated displayed time of the day |
|  |  | Hand hygiene | Considered satisfactory when there is running water and soap for handwashing or alcohol handrub |
|  |  | Cough hygiene | Considered satisfactory when patients are informed about cough hygiene by sign boards and are provided with Surgical masks, sputum container prefilled with 5% phenol/cresol and sputum is disposed daily as per infection control guidelines |
|  |  | Wet mopping | Considered satisfactory when the floor is mopped with disinfectant liquid at least twice in a day |
|  |  | Human resources | Considered satisfactory if the Health Care Workers (HCWs) are trained in Universal work Precautions, Health Care Waste Segregation, AIC measures with particular reference to TB and HCWs posted in DR-TB ward are not immunocompromised or not on immunosuppressants |
| 1.2 | Environmental AIC measures | Ventilation | Considered satisfactory when   1. There is presence of fixed unrestricted openings forming 20% of the floor area, e.g. windows, ventilator windows which are open at all times during the day and night in all seasons and “opened” to the environment OR 2. When assisted ventilation is being used (e.g. ceiling fans, standing fans, exhausts), they should be ensured that these are kept switched on at all times. OR 3. The facility uses Ultraviolet Germicidal Irradiation (UVGI) |
| 1.3 | Personal Protective AIC measures | Personal Protective Equipment | Considered satisfactory when  1. Personal Respiratory Protection (N 95) mask is available for all the staff working in DR-TB center AND  2. All the staff are wearing at the time of visit |
| **2.** | **Patient waiting area** | | |
| 2.1 | Administarative AIC measures | Cough hygiene | Considered satisfactory when patients are informed about cough hygiene by sign boards/posters and are provided with Surgical masks/tissues and dustbins are provided for the disposal of masks/tissues. |
|  |  | Patient fastracking and separation | Considered satisfactory when TB suspects and cases should be moved to the head of the line for whatever services they want or need, e.g., laboratory, medication refills, or medical investigation and a staff person should direct or escort the patient to a separate waiting area which has the highest natural ventilation possible. |
|  |  | Wet mopping | Considered satisfactory when the floor is mopped with disinfectant liquid atleast twice in a day |
| 2.2 | Environmental AIC measures | Ventilation | Considered satisfactory when the patient waiting area there is presence of fixed unrestricted openings forming 20% of the floor area, e.g. windows, ventilator windows which are open at all times in all seasons and “opened” to the environment. When assisted ventilation is being used (e.g. ceiling fans, standing fans, exhausts), they should be ensured that these are kept switched on at all times |
| **3.** | **Chest Medicine Out patient Department** | | |
| 3.1 | Administrative AIC measures | Facility for Hand hygiene | Considered satisfactory when there is availability of either running water and soap or alcohol handrub |
|  |  | Wet mopping | Considered satisfactory when the floor is mopped with disinfectant liquid at least twice in a day |
|  |  | Human resources training | Considered satisfactory if the Health Care Workers (HCWs) are trained in AIC measures with particular reference to TB |
| 3.2 | Environmental AIC measures | Ventilation | Considered satisfactory when there is presence of fixed unrestricted openings forming 20% of the floor area, e.g. windows, ventilator windows which are open at all times in all seasons and “opened” to the environment. When assisted ventilation is being used (e.g. ceiling fans, standing fans, exhausts), they should be ensured that these are kept switched on at all times. |
| 3.3 | Personal Protective AIC measures | Personal Protective Equipment | Considered satisfactory when  1. Personal Respiratory Protection (N 95) mask is available for all the staff working in DR-TB center AND  2. All the staff are wearing at the time of visit |
| 4. | **CDST (Culture and Drug Sensitivity Testing) Laboratory** | | |
| 4.1 | Administrative AIC measures | Standard Operating Procedures | Considered satisfactory when there is a standard written protocol for all laboratory procedures from sputum handling through culture manipulation and waste disposal |
|  |  | Human resources training | Considered satisfactory if all the lab technicians are trained in AIC measures with particular reference to TB and are a part of orientation/ refresher trainings once a year |
|  |  | Signage | Considered satisfactory when signs/checklists are posted with emphasis to discarding bins/ buckets/ containers |
|  |  | Lab reports | Considered satisfactory when the lab reports/ results are timely available, monthly analysis of results and lab procedures are carried out |
|  |  | Biosafety checklist | Considered satisfactory when the following are available:   1. Biosafe centrifuge 2. Biosafe cartridges with aerosol seal buckets 3. Biosafety cabinet (Class II) with 100% exhaust (ducted outside) with annual certification and calibration 4. Safe shipping material (biosafe triple packaged containers) for transport of diagnostic specimens |
|  |  | Hand hygiene | Considered satisfactory when there is running water and soap for handwashing or alcohol handrub |
| 4.2 | Environmental AIC measures | Location and design | Considered satisfactory when   1. Laboratory is placed at the blind end of the building/ physically isolated from the common laboratory or hospital environment 2. Access to the CDST room is through an anteroom 3. The containment room where CDST is carried out is sealable in case of spill and aerosolization for decontamination |
|  |  | Restricted entry | Considered satisfactory when entry to the laboratory is restricted to laboratory personnel |
|  |  | Sterilization | Considered satisfactory when autoclave is provided within the laboratory facility |
| 4.3 | Personal Protective AIC measures | Personal Protective Equipment | Considered satisfactory when all the following are available and used at the time of visit:   1. separate clothing for lab professionals 2. closed-toe footwear 3. Personal Respiratory Protection (N 95) mask |

AIC- Airborne infection control, DR-TB- Drug resistant tuberculosis, CDST-Culture and Drug sensitivity testing

**Annex S2:** Operational definitions used to assess Knowledge about Cough Hygiene and Sputum Disposal of the patients admitted in DR-TB wards of DR-TB centers, Karnataka, India, 2016-17

| **Sl.No** | **Variable** | **Operational definitions** |
| --- | --- | --- |
| **1** | Cough hygiene | Knowledge of the patient shall be considered satisfactory if the patient answers the first response correctly and any one of the other two responses.   1. Cover the mouth and nose with hand/ tissue when coughing or sneezing 2. Perform hand hygiene (e.g., hand washing with soap and water, alcohol-based hand rub, or antiseptic hand wash) after having contact with respiratory secretions and contaminated objects/materials. 3. Use the nearest waste basket to dispose of the tissue after use |
| **2** | Purpose of use of surgical masks | Satisfactory answer to this question shall be to restrict the aerosols/ droplets release or to prevent the spread of Tuberculosis |
| **3** | Situations that demand mask usage | Satisfactory response to this question is that the mask is to be used all the time during the stay in the hospital |
| **4** | Disposal of sputum | Satisfactory response shall be that sputum needs to be spitted into the container with lid containing a disinfectant solution, emptied everyday in the toilet and flushed |

**Annex S3:** Interview guides used for key informant interviews at Drug Resistant Tuberculosis (DR-TB) centres of Karnataka, India, 2016-17

**Staff type: Administrator**

DR-TB Facility Name: Date of the Interview:

Method of Interview: Duration of the interview:

1. Can you please share with me about the IC committee of your hospital
2. What are the components of AIC addressed in infection control guidelines of your hospital?
3. Can you please share with me the challenges that you face from the availability of resources point of view? For example, can you share the different kinds of resources that you know you will need.
4. First, human resources (Probe for examples)
5. Second, Finances/money resources (Probe for examples)
6. Third, improvements in infrastructure and materials (Probe for examples)
7. Do you feel you have any problems or issues as far as the current structure of the MDR Centre ward is concerned in terms of its location or design?
8. a. According to you, what are the roles of the **doctors** in making the MDR centre compliant with AIC measures (Probe for examples)

b. According to you, what are the roles of **nurses** in making the MDR centre compliant with AIC measures (Probe for examples)

c. According to you, what are the roles the **housekeeping** staff in making the MDR centre compliant with AIC measures (Probe for examples)

d. What skills do you feel these categories of staff (doctors, nurses, housekeeping staff) are presently lacking to help the facility comply with AIC guidelines?

*[Probes: Knowledge and attitudes regarding AIC, his/her leadership style, emotional intelligence regarding Housekeeping staff (self-awareness, social awareness, self-management, vital nature of AIC in the MDR TB facility), communication with student, colleagues and community, Change management]*

*[Prompt: Expect responded to mention – recruitment issues, retaining staff, training]*

1. According to you, what other measures you want to suggest to reduce the transmission of the disease in the hospital setting?
2. Can you share some of the “best practices” which are presently incorporated in this DR-TB centre for airborne infection control.

**Debriefing** – Mention some of the key points mentioned by the participants **to check your understanding** and thank the participant for their valuable information

**Staff type: Doctors**

DR-TB Facility Name: Date of the Interview:

Method of Interview: Duration of the interview:

1. How long have you been working here? Can you share with us your daily routine at work?
2. Can you share with us what training/orientation you received before your began working here in this ward?
3. How do you feel about your present working conditions?
4. How do you feel about the housekeeping staff and the work that they do on a daily basis?
5. How do you feel about the nurses and the work that they do on a daily basis?
6. Do you feel you have all the resources you need to do your job well? (Why/Why not?)
7. What are you using **now at present** in your normal course of daily work with regard to personal protection?
8. In your knowledge, what are the personal protective devices which you **need to wear** or use when you are working in this specific ward?
9. Can you share with us the reasons **why you do wear** these protective devices?
10. Can you share with us the reasons **you do not wear** these protective devices?
11. Could you please explain how sputum is **disposed** of in this MDR TB facility from the patients’ bedside?
12. Can you share with us the routine procedures you follow when a new patient is admitted to this ward?
13. What are the general measures you **would like to take** to prevent the transmission of this disease?
14. Can you share some of the “best practices” which are presently incorporated in this DR-TB centre for airborne infection control.

**Staff type: Staff nurses**

DR-TB Facility Name: Date of the Interview:

Method of Interview: Duration of the interview:

1. How long have you been working here? Can you share with us your daily routine at work?
2. Can you share with us what training/orientation you received before your began working here in this ward?
3. How do you feel about your present working conditions? (Probe specifically to this question: Get opinions from nurses about their roster, duration of time, posting, working hours, shifts)
4. What are the hazards for the other patients/relatives of patients who visit this hospital/ward because of this present set of admitted patients?
5. How do you feel about the senior staff and the work that you do on a daily basis?
6. Do you feel you have all the resource you need to do your job well? (Why/Why not?)
7. What are you using **now at present** in your normal course of daily work with regard to personal protection?
8. In your knowledge, what are the personal protective devices which you **need to wear** or use when you are working in this specific ward?
9. Can you share with us the reasons **why you do wear** these protective devices?
10. Can you share with us the reasons **you do not wear** these protective devices?
11. Could you please explain how sputum is **disposed** of in this MDR TB facility from the patients’ bedside?
12. Can you share with us the routine procedures you follow when a new patient is admitted to this ward?
13. What are the general measures you **would like to take** to prevent the transmission of this disease?

**Staff type: Housekeeping staff**

DR-TB Facility Name: Date of the Interview:

Method of Interview: Duration of the interview:

1. How long have you been working here? Can you share with us your daily routine at workplace.
2. Can you share with us what training/orientation you received before you began working here in this ward?
3. How do you feel about your present working conditions?
4. Are you aware of the nature of the disease that the patients here are facing?
5. How do you feel about the senior staff and the work that you do on a daily basis?
6. Do you feel you have all the resource you need to do your job well? (Why/Why not?)
7. What are you using **now at present** in your normal course of daily work with regard to personal protection?
8. In your knowledge, what are the personal protective devices which you **need to wear** or use when you are working in this specific ward?
9. Can you share with us the reasons you do not wear these protective devices?
10. Could you please explain how sputum is **disposed** of in this MDR TB facility from the patients’ bedside?

**Debriefing** – Mention some of the key points mentioned by the participants **to check your understanding** and thank the participant for their valuable information
